# Predictive and Prognostic Biomarkers in Pediatric Intussusception—A Systematic Review

**DOI:** 10.3390/jcm15083114

**Published:** 2026-04-19

**Authors:** Kristina Jurković, Karla Pehar, Danijela Jurić, Marko Bašković

**Affiliations:** 1 School of Medicine, Catholic University of Croatia, Ilica 242, 10000 Zagreb, Croatia; 2 Department of Pediatric Surgery, Children’s Hospital Zagreb, Ulica Vjekoslava Klaića 16, 10000 Zagreb, Croatia; 3 School of Medicine, University of Zagreb, Šalata 3, 10000 Zagreb, Croatia; 4 Scientific Centre of Excellence for Reproductive and Regenerative Medicine, School of Medicine, University of Zagreb, Šalata 3, 10000 Zagreb, Croatia; 5 Croatian Academy of Medical Sciences, Kaptol 15, 10000 Zagreb, Croatia

**Keywords:** intussusception, invagination, biomarkers, prognostic, predictive, children, pediatrics, pediatric surgery

## Abstract

**Background/Objectives**: Pediatric intussusception, a condition where part of the intestine telescopes into an adjacent segment, predominantly affects children aged 6–18 months. Prompt diagnosis and management are crucial to prevent serious complications such as ischemia or necrosis. This systematic review aims to comprehensively evaluate and synthesize existing research on predictive and prognostic biomarkers associated with pediatric intussusception that can aid in early diagnosis, severity assessment, outcome prediction, and treatment. **Methods**: A comprehensive literature search was conducted across PubMed, Scopus, and Web of Science using specific MeSH and free-text terms related to intussusception, biomarkers, and the pediatric population. The review followed PRISMA guidelines, with independent screening, data extraction, and quality assessment using the Joanna Briggs Institute critical appraisal tools. A total of 47 studies, mostly retrospective cohorts from diverse countries, with over 20,000 patients, were included. **Results**: The studies identified numerous biomarkers associated with disease severity, including hematological markers and indices (e.g., WBC counts and neutrophil-to-lymphocyte ratio), inflammatory markers (CRP and cytokines), biochemical markers (serum lactate, D-dimer, and electrolytes), and novel molecular markers (I-FABP, MCP-1, and transfer RNA fragments). Elevated inflammatory markers and derived ratios consistently predicted bowel necrosis, ischemia, and need for surgery. Biochemical markers like serum lactate and D-dimer correlated with ischemic severity. Emerging molecular biomarkers show promise for early, non-invasive risk stratification. However, heterogeneity in study designs, assay methods, and cutoff values currently limits immediate clinical application. **Conclusions**: Biomarker research offers valuable tools for improving pediatric intussusception management, with the potential to enhance early diagnosis and outcome prediction. While traditional markers are useful, novel molecular and protein biomarkers hold promise for more specific and rapid assessment. Validation through multicenter, prospective studies and standardized protocols is essential before routine implementation. Integrating biomarkers with clinical and imaging data could refine decision-making, ultimately reducing morbidity and improving prognosis in affected children.

## 1. Introduction

Intussusception in children is a condition where part of the intestine telescopes into an adjacent segment. It most commonly affects children aged 6 to 18 months and is more frequent in males [1]. Symptoms include episodic abdominal pain that waxes and wanes, vomiting, abdominal bloating, and bloody stool [2]. The causes of intussusception in children are largely unknown, with approximately 90% of cases arising from an idiopathic origin. When causes are identified, they may include infections such as viral gastroenteritis, anatomical factors like Meckel’s diverticulum or intestinal tumors, lymphoid hyperplasia, and altered intestinal motility [3,4]. Diagnosis is primarily achieved through ultrasound imaging [5]. Intussusception is not usually immediately life-threatening and is often successfully treated with non-surgical methods. The most common approach involves the use of barium, water-soluble contrast, or air-contrast enemas, which serve both to confirm the diagnosis and to reduce the intussusception. The success rate of enema reduction exceeds 80%. However, there is a possibility of recurrence, with up to 10% of cases reoccurring within 24 h after initial treatment [3,6,7]. The longer the prolapsed intestinal segment remains reduced and the longer it goes without an adequate blood supply, the less effective the non-surgical reduction becomes. In cases where non-surgical reduction fails or is not feasible, surgical intervention becomes necessary. If attempts at manual reduction are unsuccessful, the affected section of the intestine may need to be surgically removed. Additionally, a laparoscopic approach can be employed, where the surgeon uses forceps to gently pull apart the intestinal segments and restore normal anatomy [8,9,10]. Prompt diagnosis and treatment are crucial to prevent complications such as bowel ischemia or perforation. Overall, with timely intervention, the prognosis for children with intussusception is excellent [1,2,11]. Overall, intussusception remains a significant pediatric emergency but is highly treatable with prompt diagnosis and management.

Biomarkers are measurable indicators of biological processes, pathological conditions, or responses to therapeutic interventions, playing a crucial role in disease diagnosis, prognosis, and monitoring. They can include molecules such as proteins, nucleic acids, or metabolites that are detected in blood, tissues, or other bodily fluids [12]. Biomarkers for intestinal conditions in children are vital for early diagnosis, guiding treatment decisions, and monitoring disease activity. In inflammatory disorders such as inflammatory bowel disease (IBD), fecal calprotectin and serum C-reactive protein (CRP) serve as reliable indicators of intestinal inflammation and mucosal injury [13]. Markers of intestinal permeability, like zonulin, provide insights into mucosal barrier integrity, which can be compromised in various gastrointestinal conditions [14]. Beyond inflammatory diseases, biomarkers are also being explored for surgical pathologies such as intussusception, volvulus, and necrotizing enterocolitis (NEC) [15,16,17]. For example, elevated inflammatory cytokines, specific imaging biomarkers, and laboratory parameters can assist in early detection and assessment of severity, potentially guiding surgical interventions. Additionally, emerging markers such as microRNAs and metabolomic profiles show promise in diagnosing and differentiating among conditions like congenital malformations, infectious enterocolitis, and short bowel syndrome [18,19]. Advancements in biomarker research are enhancing the ability to diagnose, stratify, and personalize treatment for a broad spectrum of pediatric intestinal diseases, ultimately improving clinical outcomes.

This systematic review aims to comprehensively evaluate and synthesize existing research on predictive and prognostic biomarkers associated with pediatric intussusception. The review seeks to identify biomarkers that can aid in early diagnosis, stratify disease severity, predict disease outcomes, and guide therapeutic decision-making to improve patient management and prognosis in affected children.

## 2. Materials and Methods

### 2.1. Study Design and Search Strategy

A systematic review was performed according to Preferred Reporting Items for Systematic Reviews and Meta-Analysis (PRISMA) guidelines. The review protocol was registered with the International Prospective Register of Systematic Reviews (PROSPERO, CRD420251268322). The PRISMA 2020 Checklist is included in the Supplementary Materials [20].

To identify the total number of articles of interest, we searched the electronic databases PubMed, Scopus, and Web of Science on 01 January 2026. Reading the articles and processing the data took one month. The search combinations used included the Boolean operators “AND” and “OR” in combination with the following MeSH and free-text terms: [(intussuscept*) OR (invaginat*) OR introversion OR telescoping OR (infold*)] AND [bowel OR gut OR (intestin*)] AND [(predict*) OR (prognos*) OR (forecast*) OR (outcome*)] AND [(laborator*) OR blood OR hematology OR haematology OR plasma OR serum OR acid-base OR urine OR (factor*) OR (test*) OR (indicator*) OR (marker*) OR (biomarker*)] AND [(pediatr*) OR (paediatr*) OR (child*) OR kids].

The Boolean logical operator expressions were used to search within databases, as follows:

PubMed: (“intussuscept*”[All Fields] OR “invaginat*”[All Fields] OR “introversion”[All Fields] OR “telescoping”[All Fields] OR “infold*”[All Fields]) AND (“bowel”[All Fields] OR “gut”[All Fields] OR “intestin*”[All Fields]) AND (“predict*”[All Fields] OR “prognos*”[All Fields] OR “forecast*”[All Fields] OR “outcome*”[All Fields]) AND (“laborator*”[All Fields] OR “blood”[All Fields] OR “hematology”[All Fields] OR “haematology”[All Fields] OR “plasma”[All Fields] OR “serum”[All Fields] OR “acid-base”[All Fields] OR “urine”[All Fields] OR “factor*”[All Fields] OR “test*”[All Fields] OR “indicator*”[All Fields] OR “marker*”[All Fields] OR “biomarker*”[All Fields]) AND (“pediatr*”[All Fields] OR “paediatr*”[All Fields] OR “child*”[All Fields] OR “kids”[All Fields]).

Scopus: TITLE-ABS-KEY ((((intussuscept*) OR (invaginat*) OR introversion OR telescoping OR (infold*)) AND ((bowel OR gut OR (intestin*)) AND ((predict*) OR (prognos*) OR (forecast*) OR (outcome*)) AND (laborator*) OR blood OR hematology OR haematology OR plasma OR serum OR acid-base OR urine OR (factor*) OR (test*) OR (indicator*) OR (marker*) OR (biomarker*)) AND ((pediatr*) OR (paediatr*) OR (child*) OR (kids))).

Web of Science (editions: A&HCI, BKCI-SSH, BKCI-S, CCR-EXPANDED, ESCI, IC, CPCI-SSH, CPCI-S, SCI-EXPANDED, SSCI): ((intussuscept*) OR (invaginat*) OR (introversion) OR (telescoping) OR (infold*)) AND ((bowel OR gut OR (intestin*)) AND ((predict*) OR (prognos*) OR (forecast*) OR (outcome*)) AND (laborator*) OR blood OR hematology OR haematology OR plasma OR serum OR acid-base OR urine OR (factor*) OR (test*) OR (indicator*) OR (marker*) OR (biomarker*)) AND ((pediatr*) OR (paediatr*) OR (child*) OR kids) (All Fields).

**Figure 1:**
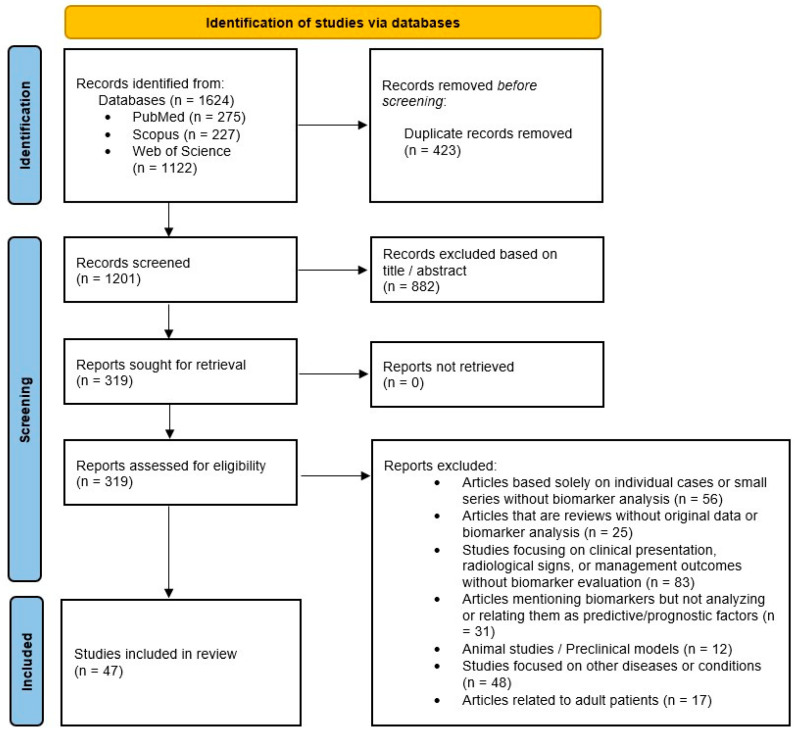
PRISMA flow diagram.

No filters were used during the search, nor were limits set regarding article type and language. Importantly, all included manuscripts were peer-reviewed articles, ensuring a standard of scientific rigor and quality. The search strategy was designed to be comprehensive yet specific, with the use of both controlled vocabulary and free-text keywords, and the Boolean operators were carefully structured to link biomarker-related terms with disease concepts, thereby minimizing the inclusion of unrelated laboratory studies. No text analysis tools or artificial intelligence algorithms were employed during the search or screening processes. The study selection process is described in Figure 1.

### 2.2. Inclusion and Exclusion Criteria

To be included, the study had to contain data on patients aged between 0 and 18 years diagnosed with or treated for intussusception and in whom at least one prognostic or predictive biomarker was evaluated or included in the analysis, regardless of whether the study additionally dealt with clinical, radiological, and other indicators. Studies were excluded if they did not involve patients aged 0 to 18 years with diagnosed or treated intussusception; lacked assessment of prognostic or predictive biomarkers; were case reports, reviews, conference abstracts, editorials, book chapters, commentaries or debate reports; involved non-human subjects; contained insufficient or unusable data (e.g., missing key variables or incomplete datasets); or were not published in peer-reviewed journals. The following information was sought within the studies: patients’ age, patients’ gender, sample size, types of intussusceptions, causes of intussusception, type of biomarker, method of biomarker measurement, timing of biomarker assessment, predictive or prognostic outcomes, treatment modalities used, the association between biomarker and outcomes, and threshold or cutoff values used for positivity.

### 2.3. Screening Process, Critical Appraisal and Data Extraction

After removing duplicate records, studies were selected through a four-step process. First, titles and abstracts were independently screened by three investigators (K.J., K.P., and D.J.) who were blinded to each other’s assessments. Before screening, the investigators underwent training sessions to calibrate their understanding of the predefined inclusion and exclusion criteria, which were clearly specified in the protocol. Inter-rater reliability was assessed using Cohen’s kappa statistic to ensure consistency during the initial screening phase (κ = 0.78, 95% CI: 0.65–0.91). In the second step, articles that met the preliminary criteria based on title and abstract were retrieved in full text. These full-text articles were reviewed independently by the same investigators, with blinding maintained to reduce bias. Disagreements between reviewers were discussed during a consensus meeting involving a fourth investigator (M.B.), where unresolved conflicts were documented and resolved through discussion until consensus was reached. Subsequently, the aforementioned data were extracted by the investigators, including reasons for exclusion during full-text review (see flow diagram summarizing the selection of studies for inclusion in the systematic review). The review protocol was registered with the International Prospective Register of Systematic Reviews (PROSPERO, CRD420251268322).

### 2.4. Assessment of the Methodological Quality and the Risk of Bias of Studies

Depending on the type of study, the methodological quality, and potential sources of bias in the included studies, they were independently assessed by K.J., K.P., D.J., and M.B. using the Joanna Briggs Institute (JBI) Critical Appraisal Checklists for Cohort Studies, Cross-sectional Studies, and Case–Control Studies [21] (Table S1). Disagreements between the investigators at various stages of the review were resolved through discussion. For scoring, each ‘Yes’ response was awarded one point, while ‘No’, ‘Unclear’, and ‘Not applicable’ responses received zero points. The total score was determined by summing the points from all ‘Yes’ responses and was then converted into a percentage by dividing by the maximum possible score. Based on this percentage, the methodological quality of each study was classified as low (<50%), moderate (50–74%), or high (>75%).

## 3. Results

### 3.1. Study Selection

Based on the described search strategy, a total of 1624 records were identified across the PubMed (275), Scopus (227), and Web of Science (1122) databases. Following deduplication, 423 duplicate records were removed prior to the screening process. During the initial screening based on titles and abstracts, 882 records were excluded. Subsequently, 319 articles remained, of which 272 were excluded based on predefined inclusion and exclusion criteria. Ultimately, 47 studies met the eligibility criteria and were included in this systematic review. All included studies were original research articles employing retrospective or prospective study designs. The PRISMA flow diagram illustrating the literature selection process is presented in Figure 1.

### 3.2. Study Characteristics, Risk of Bias, and Summary of Included Studies

Upon assessing the methodological quality and risk of bias using the JBI Critical Appraisal Checklist for Cohort Studies, Cross-sectional Studies, and Case–Control Studies, 8 (17%) studies were classified as high quality, 33 (70.2%) as moderate quality, and 6 (12.8%) as low quality based on the overall quality assessment score (Table S1). The main risks of bias across the studies include unclear or unvalidated measurement of exposures and outcomes, insufficient control of confounding factors, and incomplete follow-up or inadequate reporting of follow-up procedures.

The most frequently represented countries from which the studies originate are China, which contributes a significant proportion of the studies, followed by Turkey, South Korea, and Taiwan. The geographic distribution highlights the wide international interest and research activity in the field of biomarkers in pediatric intussusception. The study designs across the included research are varied, with the majority being retrospective cohort studies. Based on the data from all 47 studies, the total sample size exceeds 20,000 patients, with individual study sizes ranging from as few as 18 to over 10,000 participants. The patients’ ages vary widely, from infants around 1 month old to late childhood, with many studies reporting mean ages between 6 months and 3 years. The gender distribution is generally balanced, though specific ratios differ across studies. Of the studies reporting types and causes, the most common type of intussusception observed was ileocolic. Most cases were idiopathic, while in some patients, pathological lead points were found. The main characteristics of the studies included in this systematic review are shown in Table 1.

### 3.3. Hematological and Blood Parameters

Hematological parameters have been extensively studied for their prognostic value in pediatric intussusception. The white blood cell (WBC) count is often associated with the severity and outcome of intussusception, with elevated WBC levels (>10 × 10^9^/L and >20 × 10^9^/L) serving as significant predictors of surgical intervention, bowel necrosis, and failure of reduction. The neutrophil count similarly correlates with adverse outcomes, with neutrophilia being associated with an increased risk of bowel necrosis, ischemia, and the need for surgery. The neutrophil-to-lymphocyte ratio (NLR) has emerged as a valuable inflammatory marker, with higher NLR values (>1.2, >4.52, or >5.72) associated with an increased likelihood of bowel necrosis, the need for surgery, and resection. Thresholds such as NLR > 4.52 and neutrophils >9420/cc have shown good sensitivity and specificity for predicting surgical intervention and bowel ischemia. Overall, elevated WBC, neutrophil count, and NLR are consistent indicators of more severe disease progression and poorer prognosis in pediatric intussusception [23,26,30,31,35,36,38,39,43,44,47,51,52,54,64,66,68]. Elevated monocyte ratios are strongly associated with early recurrence of intussusception, indicating their potential as a predictive marker for relapse. Increased platelet counts, when combined with other factors, help predict treatment success and the presence of intestinal necrosis. Notably, a platelet-to-lymphocyte ratio (PLR) greater than 188.5 serves as a significant threshold, with high sensitivity and specificity, to identify patients at risk of intestinal necrosis and in need of surgical intervention [30,42,47,52]. Low hemoglobin levels, specifically below 12.2 g/dL, are significantly associated with failure of pneumatic reduction in children with ileocolic intussusception. Anemia markedly increases the likelihood of unsuccessful non-surgical reduction procedures, highlighting hemoglobin as a key prognostic biomarker in this setting [54,60].

### 3.4. Markers of Inflammation and Immune Response

Elevated CRP levels are strongly associated with worse clinical outcomes in pediatric intussusception, including the need for surgical intervention, bowel resection, and the presence of intestinal necrosis. Studies identify cutoff values ranging from approximately 3.4 mg/dL to over 11 mg/dL that can predict the likelihood of these complications with varying sensitivity and specificity. Higher CRP levels also correlate with increased inflammation and severity, making it a useful biomarker for risk stratification and guiding treatment decisions in affected children [28,31,32,33,34,35,36,37,38,39,44,47,52,58,62,63,65]. Elevated levels of IL-6 are consistently associated with disease activity and severity in ileal lesions, with IL-6 levels above 1.6 pg/mL serving as a key cutoff for positivity. Higher IL-6 levels correlate with worse outcomes and active disease, while decreases in these markers are linked to recovery. Other cytokines, including IL-2, IL-4, IL-10, and TNF-α, have been measured, with findings indicating that higher serum concentrations are associated with more severe tissue damage and worse outcomes [33,63,65]. Serum neopterin, a macrophage activation marker, also shows increased levels in complicated cases, indicating immune activation during severe intussusception [65]. Additionally, chemokines like MCP-1 are elevated in cases with early recurrence, serving as potential markers for relapse risk [48]. Serum endotoxins, signaling bacterial translocation due to mucosal compromise, are elevated in severe cases, further highlighting the immune response’s role in disease progression [65]. Neuro-immune peptides such as substance P and vasoactive intestinal peptide (VIP) have also demonstrated altered serum levels, suggesting neuro-immune interactions involved in the pathogenesis and severity of intussusception [56].

### 3.5. Derived Ratios and Inflammatory Indices

Various derived ratios and indices have demonstrated high prognostic value in pediatric intussusception. The neutrophil-to-lymphocyte ratio (NLR) is among the most studied [26,27,28,30,35,44,52]. The platelet-to-lymphocyte ratio (PLR) also correlates with disease severity, with higher ratios associated with bowel ischemia and necrosis [30,52]. Low LCR values (below approximately 0.12 to 0.9) are strongly associated with an increased risk of intestinal necrosis and the need for bowel resection. An LCR cutoff of 0.1233 predicts the need for surgery with high sensitivity and specificity. Patients with LCR below this threshold are more likely to require resection due to necrosis [27,32,35,52]. The systemic immune-inflammation index (SII), calculated from platelets, neutrophils, and lymphocytes, demonstrates remarkable sensitivity for predicting surgical needs [25]. An elevated CAR is significantly associated with the need for intestinal resection. A CAR value above 7.73 predicts a higher likelihood of necrosis, with high sensitivity and specificity. Each unit increase in CAR increases the risk of requiring surgery due to necrosis [27,35,52]. The HALP score, comprising hemoglobin, albumin, lymphocytes, and platelets, inversely correlates with disease severity, with lower scores indicating higher risk for complicated cases [30].

### 3.6. Biochemical and Metabolic Parameters

Serum biochemical parameters provide valuable prognostic information in pediatric intussusception. Electrolyte disturbances, notably hyponatremia and hypokalemia, are common in severe cases and are independently associated with increased risks of bowel ischemia, perforation, and necrosis. Hyponatremia (serum sodium < 135 mmol/L) is strongly associated with major surgical complications in children with intussusception, making it a key prognostic indicator. Hypokalemia (serum potassium < 3.5 mmol/L) is linked to a higher risk of failure of nonoperative management and may indicate more severe disease [29,34,46]. Lower serum albumin levels (≤3.5 g/dL) are strongly associated with increased risk of intestinal ischemia, necrosis, and the need for resection. Albumin, especially when combined with markers like CRP and other inflammatory factors, significantly improves prediction accuracy for adverse intestinal outcomes [35,52]. IMA levels are significantly elevated in intussusception cases and vary according to the severity of ischemia, although with moderate sensitivity and specificity at the identified cutoff of 60.1 ng/mL [40]. Elevated lactic acid levels are significantly associated with poorer outcomes in pediatric intussusception, with higher levels indicating increased risk. Lactic acid levels ≥ 3.0 mmol/L have a high positive predictive value (88.9%) for adverse prognosis. Overall, higher lactic acid concentrations correlate with worse clinical outcomes, making it a relevant biomarker for prognosis [55]. BUN is occasionally elevated in dehydrated or systemic shock states, but its direct predictive value is limited in this context [63]. Elevated serum TBA level (≥6.98 μmol/L) is a strong independent predictor of ischemia in children with HSP, associated with increased risks of intestinal necrosis, operative intervention, and longer hospital stays, highlighting its potential utility in risk stratification and management [41].

### 3.7. Oxidative Stress and Lipid Peroxidation Markers

Oxidative stress markers such as malondialdehyde (MDA) have been investigated as indicators of tissue injury severity. Elevated MDA levels reflect increased lipid peroxidation, correlating with ischemic damage and bowel necrosis in pediatric intussusception. Although data are limited, these markers may serve as adjuncts in assessing the extent of oxidative damage and tissue viability, highlighting the role of oxidative stress in disease progression [65].

### 3.8. Serum Proteins and Peptides

Alpha-GST is a strong predictor for the management of ileocolic intussusceptions, with levels above 3.29 ng/mL indicating a higher likelihood of requiring intervention. Elevated alpha-GST is significantly associated with cases that do not resolve spontaneously, showing a high diagnostic accuracy (AUC: 0.917) and good sensitivity (88.9%) and specificity (85.7%). Therefore, measuring alpha-GST can help identify patients who may need more aggressive treatment [28]. Intestinal fatty-acid-binding protein (I-FABP) has emerged as a promising marker, with higher serum levels correlating with the extent of bowel necrosis; a cutoff of approximately 1538 ng/mL has demonstrated moderate sensitivity and specificity for predicting bowel resection [59].

### 3.9. Acid-Base Balance and Gas Exchange Parameters

Blood gas analysis reveals that acidosis, characterized by decreased blood pH and bicarbonate levels, is prevalent in severe ischemic cases. Elevated lactic acid levels are associated with tissue hypoperfusion, bowel necrosis, and failure of non-surgical management. Such parameters are critical in early assessment, guiding timely surgical intervention to prevent irreversible damage [34,55].

### 3.10. Other Biomarkers and Novel Markers

Coagulation markers like fibrinogen and D-dimer are elevated in patients with bowel ischemia due to systemic inflammatory response and hypercoagulability. Elevated D-dimer levels, particularly above 0.24 mg/L and 1 mg/L, are strongly associated with gastrointestinal complications such as intestinal necrosis and ischemia in children with IgA vasculitis and Henoch–Schönlein purpura. Fibrinogen levels exceeding 1.26 g/L independently predict intestinal necrosis in children undergoing surgical reduction for intussusception [36,39,41,50,53]. Serum CK-MB, although traditionally a cardiac marker, has been explored in the context of systemic inflammation and ischemic injury; however, evidence regarding its utility remains limited [43]. Emerging biomarkers, such as serum transfer RNA fragments (tRFs), demonstrate promising diagnostic potential; for example, elevated levels of specific tRFs correlate with bowel necrosis and could serve as novel, non-invasive biomarkers [49].

## 4. Discussion

This systematic review synthesizes current evidence on predictive and prognostic biomarkers associated with pediatric intussusception, highlighting their potential to improve early diagnosis, assess disease severity, predict outcomes, and guide management strategies. The findings indicate that a wide array of biomarkers—ranging from hematological markers and indices, inflammatory markers, and biochemical markers to emerging molecular markers—are significantly associated with critical clinical outcomes such as bowel necrosis, recurrent episodes, failure of non-surgical reduction, and the need for surgical intervention. Notably, markers of systemic inflammation, such as the neutrophil-to-lymphocyte ratio, C-reactive protein, and cytokines like IL-6, consistently demonstrate strong predictive value for disease severity and complications. Similarly, biochemical parameters, including serum lactate, D-dimer, and electrolyte disturbances, serve as valuable indicators of ischemia and systemic response. The identification of novel biomarkers such as intestinal fatty-acid-binding protein, ischemia-modified albumin, and transfer RNA fragments opens promising avenues for non-invasive, rapid risk stratification. Collectively, these biomarkers have the potential to refine clinical decision-making, facilitate timely interventions, and ultimately improve outcomes in pediatric patients with intussusception.

Despite advances in diagnostic imaging and minimally invasive treatments, the management of pediatric intussusception is still largely driven by clinical judgment and imaging findings, which may sometimes be insufficient to predict disease course or guide optimal management [69,70,71,72]. The integration of biomarkers—measurable indicators reflecting disease severity, tissue ischemia, inflammatory response, or tissue injury—offers an appealing avenue to enhance clinical decision-making [73,74].

The pathophysiology of pediatric intussusception involves complex interactions between intestinal motility, lymphoid hyperplasia, infectious triggers, and possibly genetic predispositions. As the intussuscepted bowel becomes compromised, ischemia and necrosis ensue, leading to systemic inflammatory responses [75]. Currently, clinical assessment and imaging—primarily ultrasonography—are the mainstays for diagnosis and assessment of reduction success. However, these methods have limitations: clinical signs can be nonspecific, and imaging findings may not reliably predict ischemic severity or recurrence risk [69,71,72]. Biomarkers provide an objective, rapid, and potentially quantifiable means to assess disease activity and predict outcomes. They could facilitate early stratification of patients at high risk of complications, guide the urgency and type of intervention, and optimize resource utilization [76,77]. Nonetheless, the heterogeneity of biomarker studies, variable methodologies, and inconsistent cutoff values underscore the need for critical evaluation before routine clinical application.

Markers such as CRP, WBC counts, and derived ratios (NLR, PLR, LCR) are among the most studied due to their ubiquity and ease of measurement. Their strength lies in their reflection of systemic inflammation and immune response, which are central to ischemic injury in intussusception. Elevated CRP and neutrophil counts have consistently been associated with bowel necrosis, suggesting their utility in identifying children requiring surgical intervention [28,31,32,33,34,35,36,37,38,39,44,47,52,58,62,63,65].

However, these markers are nonspecific. Elevated CRP and leukocyte counts can result from various infectious and inflammatory conditions, limiting their specificity in isolating ischemic severity solely attributable to intussusception. Moreover, their levels can be influenced by concomitant infections, dehydration, or systemic illness, confounding their interpretation [78,79,80]. The dynamic nature of inflammation also necessitates consideration of timing—serial measurements may be more informative than single values.

Derivatives such as NLR and PLR have demonstrated promising predictive capacity, integrating inflammatory cell ratios that may better reflect the balance between pro- and anti-inflammatory responses [81]. The high sensitivity and specificity reported for certain cutoff values, such as NLR > 4.5, are encouraging [44]. Nevertheless, these ratios are influenced by hematological variations unrelated to bowel ischemia, such as hematological disorders or anemia [82,83]. Standardization across laboratories and validation in diverse populations are essential before widespread adoption.

Serum lactate and D-dimer are biochemical markers with direct relevance to tissue hypoperfusion and coagulation activation, respectively. Elevated lactate levels are strongly associated with tissue hypoxia and ischemia, making them logical markers for bowel necrosis. Their predictive value for bowel necrosis and adverse outcomes has been well documented, and they are widely used in adult ischemic conditions [84,85]. Nevertheless, lactate levels can be elevated in other systemic conditions, including sepsis, hypotension, or metabolic disturbances, which may co-occur in critically ill children [86]. The timing of sampling relative to symptom onset is crucial; early lactate measurements might underestimate severity if taken before significant hypoperfusion develops [87]. D-dimer, a marker of hypercoagulability, correlates with ischemic vascular injury but lacks specificity, as elevated levels are common in infections, inflammation, and thrombotic states [88]. Their combined use, however, may enhance predictive accuracy for bowel necrosis [50,53,55].

Novel biomarkers such as I-FABP, alpha-GST, and MCP-1 have garnered interest due to their specificity for intestinal mucosal injury or ischemia [28,48,59]. I-FABP, a small cytoplasmic protein released during mucosal injury, has shown promise in early detection of bowel necrosis with moderate sensitivity and specificity. Its rapid release upon epithelial injury renders it suitable for early assessment, potentially before irreversible damage occurs [89,90]. Similarly, alpha-GST is a marker of hepatocyte and mucosal stress, with elevated levels correlating with necrosis [91]. MCP-1 and other cytokines reflect immune activation and chemokine-mediated recruitment of inflammatory cells, which are integral to ischemic injury progression [92]. While these biomarkers offer specificity advantages, their measurement often involves specialized assays, which may not be readily available in all clinical settings. Additionally, their kinetics, optimal timing, and cutoff thresholds require further validation in large, prospective pediatric cohorts.

The combination of individual biomarkers into ratios or indices aims to harness synergistic predictive information. The systemic immune-inflammation index (SII) and CRP/albumin ratio (CAR), for example, integrate inflammatory cell counts, acute-phase reactants, and nutritional status, reflecting a holistic picture of disease severity [25,27]. These composite indices have demonstrated high predictive values in preliminary studies, offering practical advantages: they utilize routinely available laboratory data and may improve risk stratification accuracy. Nonetheless, their clinical utility hinges on establishing standardized cutoff values, understanding confounding factors, and validating their predictive performance across different populations and clinical settings [93,94].

Despite the promising findings, several limitations temper the immediate clinical translation of these biomarkers. First, many studies are retrospective, with inherent biases and heterogeneity in patient populations, methodologies, and outcome definitions. Variability in assay techniques, timing of sample collection, and cutoff thresholds hampers comparability and generalizability. Second, the complex pathophysiology of intussusception and its complications implies that no single biomarker can reliably predict all clinical outcomes. Combining multiple markers into predictive models or nomograms may improve accuracy, but these models require rigorous validation and prospective testing before routine use [31,37,38,42,43]. Third, the influence of confounding factors—such as infections, dehydration, comorbidities, and systemic inflammatory states—must be carefully considered. Biomarkers like CRP and leukocyte counts are sensitive but nonspecific; thus, their interpretation must be contextualized within the broader clinical picture. Finally, logistical considerations such as assay availability, cost, turnaround time, and the need for serial measurements must be addressed. Biomarkers with rapid, point-of-care testing capabilities will be most valuable in urgent pediatric settings [74].

Currently, in clinical practice, hematological markers such as elevated WBC count, neutrophil levels, and NLR are the most consistently useful for risk assessment. Serum lactate further offers practical value in detecting tissue hypoperfusion and necrosis. While cytokines like IL-6 and MCP-1, as well as molecular markers such as I-FABP and alpha-GST, show potential for higher specificity, their use remains limited to research settings due to assay complexity and insufficient validation. Conversely, markers like CRP and D-dimer, though easily accessible, are too nonspecific to guide clinical decisions independently. Therefore, current practice should prioritize routine inflammatory and biochemical markers—WBC, neutrophil levels, NLR, lactate—integrated with clinical and imaging assessments, while molecular biomarkers await further validation before routine implementation.

The current landscape underscores the need for multicenter, prospective studies with standardized protocols to validate promising biomarkers and establish universally applicable cutoff values. The integration of biomarkers into clinical algorithms holds potential, but it must be complemented with clinical and imaging findings to optimize decision-making. The exploration of novel molecular markers, such as microRNAs, transfer RNA fragments, and serum proteomics, offers exciting prospects for non-invasive, highly specific diagnostics [95,96]. Advances in high-throughput technologies and bioinformatics could enable the development of comprehensive biomarker panels, facilitating personalized risk stratification [97]. Furthermore, longitudinal studies tracking biomarker kinetics pre- and post-intervention could elucidate their roles in monitoring disease progression, response to treatment, and recurrence risk [98]. The development of rapid, bedside assays for key markers like I-FABP and lactate would significantly enhance their clinical applicability [74,99].

## 5. Conclusions

Biomarker research in pediatric intussusception is a rapidly evolving field that promises to augment current diagnostic and prognostic tools. While traditional inflammatory and hematological markers provide valuable insights, their limitations necessitate cautious interpretation and validation. The advent of molecular and protein biomarkers offers hope for more specific and early detection of ischemic severity and complications. However, the translation of these findings into routine clinical practice requires rigorous validation, standardization, and integration within clinical workflows. Ultimately, the goal is to develop reliable, rapid, and non-invasive biomarker-based algorithms that can guide timely interventions, reduce morbidity, and improve long-term outcomes for children afflicted by this potentially life-threatening condition. Achieving this will depend on continued multidisciplinary research, technological innovation, and collaborative validation efforts.

## Figures and Tables

**Table 1:** Articles included in the systematic review.

Author(s), Year of Publication	Country	Study Design	Sample Size, Male: Female, Patients’ Age	Type of Intussusception	Cause	Type of Biomarker	Method of Biomarker Measurement	Timing of Biomarker Assessment	Predictive/Prognostic Outcomes	Treatment Modalities Used	Association Between Biomarkers and Outcomes	Threshold or Cutoff Values Used for Positivity
Soleimanpour et al., 2025 [22]	Iran	Retrospective cohort study	165 (55—underwent surgery, 110—treated non-surgically), 116:49, 2.68 ± 1.65 yrs/3.88 ± 3.51 yrs	Location of the intussusception: ileoileal (*n* = 8), ileocolic (*n* = 112), colocolic (*n* = 2), right lower quadrant (*n* = 12), right upper quadrant (*n* = 19), left lower quadrant (*n* = 3), left upper quadrant (*n* = 1), jejunojenunal (*n* = 3), jejuno-ileal (*n* = 1), multiple (*n* = 4)	NA	WBC, ESR, CRP	Blood tests	Pre-treatment	Comparison of groups, comparison of clinical and paraclinical characteristics	Hydrostatic or pneumatic reduction, surgery	Laboratory test results showed no significant differences between groups (*p* > 0.05). The study found that younger age, bloody stools, and ileocolic location were associated with a higher likelihood of needing surgery	NA
Ammar et al., 2025 [23]	Tunisia	Retrospective cohort study	156 (109—training group, 47—validation group)	Ileocolic	NA	WBC, CRP, sodium, potassium, chloride	Blood tests	At diagnosis	Likelihood of requiring surgical intervention	Hydrostatic or pneumatic reduction, surgery	The univariate analysis showed that the elevated WBC (*p* = 0.029) was associated with surgical treatment. Bloody stools (*p* = 0.033; OR = 2.61), the duration of symptoms (*p* = 0.028; OR = 1.02), and the length of the intussusception (*p* = 0.014; OR = 1.265) were identified as independent risk factors for surgical treatment	NA
Kamffer et al., 2025 [24]	South Africa	Retrospective cohort study	110 (73—attempts air enema, 37—primarily surgically managed), NA, Mdn 7 mth	Ileocolic	In three of the patients who were part of the unsuccessful group, a pathological lead point was found, including Meckel’s diverticulum and Wauch’s syndrome	CRP, white cell counts	Blood tests	At diagnosis	Identification of factors associated with fluoroscopy-guided air enema reduction outcomes in pediatric intussusception	Air enema, surgery	Unsuccessful fluoroscopy-guided air enema was significantly associated with younger age (*p* = 0.0249), dehydration (*p* = 0.0299), ascites (*p* = 0.0172), and increased outer wall intussusception diameter on ultrasound (*p* = 0.0026). No predictive value of elevated levels of CRP and white cell counts regarding the success of pneumatic reduction (*p* > 0.05)	NA
Liu et al., 2025 [25]	China	Retrospective cohort study	574 (469—air enema success group, 105—air enema failure group), 396:178, mean 22.13 mth	Ileocolic	Idiopathic	WBC, absolute neutrophil, absolute lymphocyte, absolute monocyte, platelet counts, SII, SIRI, NLR, PLR, PNR, LMR	Blood tests	Pre-treatment	Identification of SII as a predictor of air enema failure	Air enema, surgery	Threshold analysis revealed that, to the right of this inflection point (332.3), there was a significant 25% increase in the risk of air enema treatment failure for ICI per 100-unit increase in the SII (OR 1.25). In the ROC curve analysis, SII demonstrated the highest AUC of 0.8665 compared to other inflammation indices	The critical point for SII was estimated to be 721.5 (sensitivity, 86.67%; specificity, 71.00%)
Xu et al., 2025 [26]	China	Retrospective cohort study	87 (23—intestinal necrosis group, 64—control group), 58:29, NA	NA	NA	NLR, CRP	Automatic blood analyzer	Pre-treatment	Investigation of the risk factors of intestinal necrosis in children with intussusception	Air enema, surgery	NLRs were all higher in the bowel necrosis group (*p* < 0.05). The logistic regression analysis indicated that roundness (×100) (OR = 1.397) and blood flow signal (<grade 4) (OR = 0.099) were independent predictors of bowel necrosis in intussusception	NA
Elhadidi et al., 2025 [27]	Egypt	Retrospective cohort study	100 (60—nonresection group, 40—resection group), 46:54, 13.29 ± 11.31 8.4 ± 4.09 mth	NA	NA	CBC, albumin, CRP, NLR, PLR, LCR, CAR	Blood tests	Pre-treatment	The effectiveness of different combinations of inflammatory markers in predicting intestinal necrosis and the need for intestinal resection in cases of intussusception	Surgery	A statistically significantly higher mean CAR was observed among cases with resection. Conversely, the mean LCR was significantly lower in the resection group. CAR was a statistically significant predictor of the need for resection, with each unit increase in CAR increasing the risk by 1.42	LCR—cutoff point of 0.1233 (sensitivity of 85.7% and specificity of 90%), CAR—cutoff point of 7.73 (sensitivity of 92.6% and specificity of 90%). If a patient’s CAR exceeds 7.73 and if LCR is below 0.1233, they are more likely to need surgery due to necrosis
Ulusoy et al., 2025 [28]	Turkey	Prospective case–control	78 (52—intussusception group, 26—control group), 47:31, 24 mth	Ileocolic	NA	Alpha-GST, I-FABP, inflammatory markers	ELISA, blood tests	Pre-treatment	Alpha-glutathione S-transferase (alpha-GST), intestinal fatty-acid-binding protein (I-FABP), and inflammatory markers may predict spontaneous reduction in ileocolic intussusceptions	Hydrostatic reduction, surgery	Alpha-GST, NLR, and CRP levels were significantly higher in the group requiring intervention than in the spontaneous reduction group	Alpha-GST: 3.29 ng/mL (AUC: 0.917, sensitivity: 88.9%, specificity: 85.7%), CRP: 5.5 ng/mL (AUC: 0.808, sensitivity: 64.3%, specificity: 85.7%), NLR: 1.87 (AUC: 0.739, sensitivity: 75.0%, specificity: 71.4%)
Chang et al., 2025 [29]	Taiwan	Retrospective cohort study	11,111, 5348:3252, 0–3 years group—7562 cases (1.0 ± 1.0 yrs)/3–18 years group—3485 cases (8.8 ± 4.6 yrs)	NA	NA	Sodium, potassium	Blood tests	24 h before or on the day of diagnosis	Quantification of the association between dysnatremia and dyskalemia at presentation and major surgical complications in children with intussusception	Air or contrast enema, surgery	Electrolyte disturbances were prevalent: hyponatremia (9.3%), hypernatremia (1.8%), hypokalemia (4.7%), and hyperkalemia (2.8%). After adjustment, hypernatremia demonstrated the most potent association across all outcomes. Hyponatremia was independently associated with all three surgical outcomes, with more modest effect sizes (open reduction, bowel perforation or resection, peritonitis or sepsis)	Hyponatremia < 135 mmol/L, hypernatremia > 145 mmol/L, hypokalemia < 3.5 mmol/L, hyperkalemia > 5.0 mmol/L
Tuşat & Memiş, 2025 [30]	Turkey	Retrospective cohort study	78 (30—surgical group, 48—non-surgical group), 49:29, Mdn 19 mth	Surgical group: ileoileal (*n* = 3), ileocolic (*n* = 27)	NA	CBC, NLR, PLR, HALP score	Blood tests	Pre-treatment	Determination of whether the HALP score and the inflammatory markers scores differ between cases requiring surgical reduction and those not requiring surgical reduction in patients diagnosed with intussusception	Hydrostatic reduction, surgery	Higher NLR and PLR, lower HALP scores associated with surgical cases (*p* < 0.0001). However, no statistically significant difference was detected for CRP levels (*p* = 0.095)	NA
Yu et al., 2024 [31]	China	Retrospective cohort study	547 (414—non-intestinal necrosis, 133—intestinal necrosis and underwent resection), 365/182, training set = 15.81 ± 23.91 mth, validation set = 17.6 ± 26.98 mth	Ileo-colic (*n* = 263), ileo-cecal (*n* = 117), compound/complex (*n* = 95), multiple (*n* = 2), small intestinal (*n* = 70)	NA	A series of parameters from the blood	Blood tests	Pre-treatment	Predicting the risk of intestinal resection	Failed air enema, intestinal resection	Duration of symptoms (OR = 1.050, *p* < 0.001), CRP (OR = 1.021, *p* = 0.004), WBCs (OR = 1.110, *p* < 0.001), ascites (OR = 3.781, *p* < 0.001)	Duration of symptoms, C-reactive protein, white blood cells, and ascites were selected for inclusion in the nomogram, with a concordance index of 0.871
Xia et al., 2024 [32]	China	Retrospective cohort study	660 (442—no resection group, 218—bowel resection group), 302:140/143:65, Mdn 21.88 mth/15.73 mth	Location of intussusception: ascending colon (*n* = 483), transverse colon (*n* = 141), descending colon (*n* = 20), sigmoid colon (*n* = 16)	NA	Blood-based biomarkers	Blood tests	Pre-treatment	The necessity of bowel resection	Surgery	Bowel resection occurrence was linked to an extended duration of symptoms (OR = 2.14, *p* = 0.0015), the presence of gross bloody stool (OR = 8.98, *p* < 0.001), elevated C-reactive protein levels (OR = 4.79, *p* = 0.0072), lactate clearance rate (OR = 17.25, *p* < 0.001), and the intussusception location (OR = 12.65, *p* < 0.001)	A scoring system (totaling 14.02 points) was developed from the cumulative β coefficients, with a threshold of 5.22 effectively differentiating infants requiring bowel resection, CRP > 8.0 mg/L, LCR < 0.121
Wei et al., 2024 [33]	China	Retrospective cohort study	18 (6—routine nursing group, 12—rehabilitation training group), 11:7, 5.24 ± 3.05 yrs/5.16 ± 3.14 yrs	NA	NA	GAS, MTL, IL-2, IL-4, IL-6, IL-10, CRP, TNF-α (serum)	Radioimmunoassay, ELISA	Post-treatment—after 5 days of rehabilitation	Effects of different rehabilitation methods on gastrointestinal function and inflammatory factor levels	Surgery (laparoscopic), rehabilitation	GAS (r = 0.490) and MTL (r = 0.714) levels were positively correlated with postoperative rehabilitation (*p* < 0.05). IL-2 (r = −0.782), IL-4 (r = −0.871), IL-6 (r = −0.971), IL-10 (r = −0.979), CRP (r = −0.981), and TNF-α (r = −0.921) levels were negatively correlated with postoperative rehabilitation (*p* < 0.05)	NA
Shah et al., 2024 [34]	India	Prospective cohort study	110 (reduced and not reduced group), 72:38, mean age 13.54 months	Ileocolic	NA	CBC, CRP, blood gas analysis with serum electrolytes	Blood tests	At diagnosis	Assessment of the clinical and radiological predictors of success or failure of nonoperative management of intussusception	Hydrostatic reduction, surgery	Elevated TLC, CRP, lactate, and low potassium were significantly associated with increased failure risk (*p* < 0.001)	NA
Budiananti et al., 2024 [35]	Indonesia	Cross-sectional study	36, 24:12, mean 7 mth (no intestinal necrosis), 8 mth (intestinal necrosis)	NA	NA	WBC, lymphocytes, neutrophils, platelets, albumin, CRP, PLR, CAR, NLR, LCR	Blood tests	At diagnosis	Prediction of intestinal ischemia and necrosis	Surgery	Significantly related markers were albumin (*p* = 0.00; AUC 0.888), CRP (*p* = 0.00; AUC 0.948), CAR (*p* = 0.00; AUC 0.914), NLR (*p* = 0.032; AUC 0.714), and LCR (*p* < 0.001; AUC 0.906).	Albumin ≤ 3.5 g/dL CRP ≥ 3.37 mg/dL CAR > 1 NLR > 1.2 LCR ≤ 0.9
Mu et al., 2024 [36]	China	Retrospective cohort study	28 (21—intussusception, 7—intestinal perforation), 10:18, mean 7.2 yrs	Ileoileal (*n* = 12), ileo-colonic (*n* = 6), jejunal-jejunal (*n* = 2), colo-colonic (*n* = 1)	Associated with IgA vasculitis	Leukocytes, CRP, D-dimer	Blood tests	At diagnosis	Surgical complications in children with IgAV	Conservative treatment methods, surgery	Increased leukocytes were observed in 60.7% of children. CRP and D-dimer were elevated in 53.3% and 75% of children, respectively	NA
Liu et al., 2024 [37]	China	Retrospective cohort study	1041 (852—successful reduction group, 189—failed reduction group, 728—training set, 313—validation set), 696:345, 33.50 ± 20.98 mth/26.55 ± 27.56 mth	NA	Nested position: right colon (*n* = 818), left colon (*n* = 223)	WBC, CRP	Blood tests	At diagnosis	Development and validation of a nomogram for predicting the need for surgical intervention in pediatric intussusception after pneumatic reduction	Pneumatic reduction, surgery	Logistic regression analysis of the training set identified age, time of abdominal pain, presence or absence of hematochezia, C-reactive protein value from blood test on admission (OR 1.034, *p* < 0.001), and nested position indicated by B-ultrasound as independent predictors of intussusception intervention	NA
Liu et al., 2024 [38]	China	Retrospective cohort study	2406 (208—recurrent intussusception group, 2198—control group), 1620:786, mean 30.12 ± 19.97 mth	Excluded ileal intussusception from analysis	NA	WBC, CRP	Blood tests	Pre-treatment	Development and validation of a nomogram for predicting recurrent intussusception in children within 48 h after pneumatic reduction in primary intussusception	Pneumatic reduction	Age, abdominal pain time, white blood cell counts (OR = 1.12), and hypersensitive C-reactive protein levels (OR = 1.16) were identified as predictors and incorporated into the nomogram	NA
Mu, 2024 [39]	China	Retrospective case–control study	192 (32, 25—intussusception, 7—intestinal perforation, 160—control group), 99:93, mean 6.44 ± 2.21 yrs	Ileum-ileal (*n* = 16), ileo-colonic (*n* = 6), jejunum-jejunum (*n* = 2), colon-colic (*n* = 1)	Small bowel polyps (*n* = 2), Meckel’s diverticulum (*n* = 1), malrotation (*n* = 1)	WBC, CRP, D-dimer, erythrocyte sedimentation rate	Blood tests	At diagnosis	A summary of the clinical features of IgAV complicated by intussusception and intestinal perforation, and explore its risk factors	Enema reduction, surgery	Higher WBC, CRP, and D-dimer levels are associated with surgical complications. Multivariate logistic regression analysis indicated that age ≤ 7 years, GI symptoms prior to skin purpura, abdominal pain intensity, and timing of glucocorticoid treatment were independent risk factors of IgAV with intussusception and intestinal perforation	WBC > 10 × 10^9^/L CRP > 8 mg/L D-dimer > 0.24 mg/L
Kocaoğlu et al., 2024 [40]	Turkey	Case–control study	70 (36—intussusception group, 34—control group), 36:34, Mdn 34 mth/30 mth	Ileoileal (*n* = 9), ileocecal (*n* = 24), colocolic (*n* = 3)	NA	IMA	ELISA	Pre-treatment	Determination of the sensitivity of IMA and the correlation between IMA and the severity of intestinal ischemia in intussusception cases	Hydrostatic reduction, surgery	The mean IMA level of the intussusception group was 179.13 ± 220.33 ng/mL, whereas the mean level was found as 89 ± 70.9 ng/mL in the control group (*p* = 0.023). When the patients were categorized as ileoileal, ileocecal, and colocolic, the mean IMA levels were detected as 235.65 ± 268.14 ng/mL, 174.46 ± 212.8 ng/mL, and 46.95 ± 19.56 ng/mL, respectively	The sensitivity and specificity rates were determined as 55.6% and 55.9% using a cutoff value of 60.1 ng/mL for IMA
Yu et al., 2023 [41]	China	Retrospective cohort study	708 (613—HSP, 95—HSP with AI), HSP without AI group—357/256, Mdn 5 yrs 8 mth, HSP with AI group—56/39, Mdn 6 yrs 5 mth	According to imaging manifestations - ileocolic (*n* = 45), small intestinal (*n* = 37)	NA	Total bile acid (serum)	Blood tests	Pre-treatment (at diagnosis during GI symptoms)	High serum TBA predicts AI; higher TBA levels were associated with increased operative treatment, intestinal necrosis, and longer hospital stays	Air enema, intestinal resection	Vomiting (OR = 396.492, *p* < 0.001), haematochezia (OR = 87.436, *p* = 0.001), TBA (OR = 16.287, *p* < 0.001), and D-dimer (OR = 5.987, *p* = 0.003) were independent risk factors for abdominal-type HSP with AI	TBA > 3 μmol/L for predicting AI in children with abdominal-type HSP, TBA ≥ 6.98 μmol/L associated with an increased incidence of operative treatment, intestinal necrosis, and length of hospital stay
Yang et al., 2023 [42]	China	Retrospective cohort study	869 (787—with no relapse, 82—recurrent intussusception), 591/278, <1 year old = 312, ≥1 year old = 557	NA	NA	Blood-based biomarkers	Blood tests	Pre-treatment	Likelihood of early recurrence of intussusception (<48 h)	Enema therapy, surgery	Age (OR 7.67, *p* = 0.001), vomiting (OR 0.17, *p* < 0.001), bloody stool (OR 0.14, *p* = 0.01), and monocyte ratio (OR 9.52, *p* = 0.003) were independently associated with the clinical endpoints	Children older than 1 year in age, who lacked vomiting and bloody stool symptoms, and who exhibited an elevated ratio of monocytes, were more likely to relapse early
Zhuang et al., 2023 [43]	China	Retrospective cohort study	199 (139—training group, 60—validation group), 146:53,	Location: right side (*n* = 145), left side (*n* = 54)	NA	WBCs, platelets, CRP, fibrinogen, CK-MB, potassium, sodium, chloride	Blood tests	At diagnosis	Development and validation of a nomogram for predicting surgical intervention in pediatric intussusception after hydrostatic reduction	Hydrostatic reduction, surgery	Duration of symptoms, bloody stools, WBCs (OR = 1.203, *p* = 0.026), CK-MB (OR = 1.034, *p* = 0.040), long-axis diameter, poor prognostic signs by ultrasound and mental state were identified as the independent predictors of surgical intervention for intussusception	A model that incorporated the independent predictors was developed and presented as a nomogram. The C-index of the nomogram in the validation set was 0.948
Delgado-Miguel et al., 2023 [44]	Spain	Retrospective case–control study	511 (410—effective enema group, 101—need for surgery group), 333:178, Mdn 16 mth/16.5 mth	Ileocolic	NA	CBC, ionogram, glucose, urea, fibrinogen, CRP	Blood tests	Pre-treatment	Identification of predictors of the need for surgical treatment in ileocolic intussusception	Hydrostatic enema, surgical treatment	The surgery group presented higher median laboratory inflammatory markers: NLR (6.8 vs. 1.8; *p* < 0.001), neutrophils (10,148 vs. 7468; *p* < 0.001), and CRP (28.2 vs. 4.7; *p* < 0.001). In ROC curve analysis, NLR had an AUC of 0.925.	It was estimated that a cutoff point of NLR greater than 4.52 (sensitivity: 73.2%; specificity: 94.5%), neutrophils greater than 9420 (sensitivity: 55.4%, specificity: 82.8%), and CRP greater than 4.8 (sensitivity: 65.7%, specificity: 60.4%)
Zhang et al., 2023 [45]	China	Retrospective cohort study	624 (73—recurrence of intussusception after successful reduction with air enema), 400:224; Mdn 1.8 yrs	Site of intussusception: hepatic flexure (*n* = 237), transverse colon (*n* = 184), Ileocecal junction (*n* = 92), ascending colon (*n* = 63), splenic flexure (*n* = 31), other (*n* = 17)	NA	WBC count, neutrophil percentage, lymphocyte Percentage, CRP	Blood tests	Pre-treatment	Investigation of the factors associated with in-hospital Recurrence of intussusception	Air enema reduction	Multivariate logistic regression analysis identified age > 1 year old (OR = 7.65), secondary intestinal intussusception (OR = 14.40), and mesenteric lymph node enlargement (OR = 1.90) as factors independently associated with in-hospital recurrence of intussusception	NA
Wu et al., 2022 [46]	Taiwan	Retrospective observational study	584, 379/205, 27.2 ± 20.3 mth	Operative findings: ileocolic (*n* = 90), ileoileal (*n* = 9), ileo-ileocolic or ileo-colocolic (*n* = 39)	38 patients had pathological lead points—Meckel diverticulum (*n* = 8), bands (*n* = 8), polyp (*n* = 5), enlarged lymph nodes (*n* = 5), lymphoid mass (*n* = 5), Burkitt lymphoma (*n* = 1), enteric duplication cyst (*n* = 2), Peutz-Jeghers syndrome (*n* = 3), and cecal serosal lesions (*n* = 1)	Blood-based biomarkers	Blood tests	Pre-treatment	Prediction of the need for bowel resection	Enema reduction, surgery	Abdominal pain (OR = 0.372, *p* = 0.013), bloody stool (OR = 3.553, *p* = 0.044), and hyponatremia (OR = 4.12, *p* = 0.003) were independent predictors for surgery. Prolonged time to surgery (OR = 6.863, *p* = 0.009), long intussusception (OR = 5.088, *p* = 0.014), pathological lead point (OR = 6.926, *p* = 0.003), and ICU admission (OR = 11.777, *p* = 0.001) were associated with bowel resection	Hyponatremia (<135 mEq/L), hypochloremia (≤100 mEq/L), hyperglycemia (≥100 mg/dL)
Liu et al., 2022 [47]	China	Retrospective cohort study	8235 (5743—hydraulic enema group (HE), 2492—surgical group (SM)/398—with necrosis subgroup (SN+), 2094—without necrosis subgroup (SN−)), 3986:1757/1690:802, 1.54 ± 0.02 yrs/1.17 ± 0.04 yrs	NA	NA	74 clinical and biochemical parameters	Blood tests	Pre-treatment	Likelihood of successful reduction with an enema versus the need for surgery. Likelihood of intestinal necrosis.	Hydrostatic reduction, surgery (+/− resection)	The prediction model composed of seven variables (NEUT#, platelet, albumin, β-2MG macroglobulin, glucose, uric acid, and chlorine was suitable for HE/SM. The prediction model composed of six variables (NEUT#, average hemoglobin concentration, PLT, the total protein, CRP, and urea) was suitable for SN−/SN+	NA
Zhu et al., 2022 [48]	China	Retrospective observational study	412 (375—nonrecurring cases, 37—cases of short-term recurrence), 293:119, NA	Mass location: ascending colon (*n* = 280), transverse colon (*n* = 132)	NA	MCP-1, IL-6	ELISA	Pre-treatment	Evaluation of the relationship between the expression level of MCP-1 in peripheral blood and the short-term recurrence of primary intussusception in children	Hydrostatic reduction	Logistic regression analysis found that increased MCP-1 was a risk factor for recurrence	ROC showed that 23.24 ng/mL was used as a cutoff value (sensitivity—82.14%, specificity—75.67%)
Zhao et al., 2021 [49]	China	Cross-sectional study	40 (intussusception—20/healthy controls—20), 10:10/12:8, 30.97 ± 34.94 mth/35.21 ± 40.78 mth	Ileum colon (*n* = 16), ileum cecum (*n* = 2), ileum ileum (*n* = 2),	Predominantly idiopathic; some cases with lead points (e.g., duplication)	Transfer ribonucleic acid (tRNA)-derived fragments (tRFs) (serum)	qRT-PCR	Immediately after the final diagnosis by air enema, before reduction	Comparison of groups, focus on diagnosis rather than prognosis	Aair enema (*n* = 18), surgery (*n* = 2)	tRF-Leu-TAA-006 (0.984, *p* < 0.05), tRF-Gln-TTG-033 (0.970, *p* < 0.05), tRF-Lys-TTT-028 (0.837, *p* < 0.05) correlated with diagnosis	tRFs with AUC > 0.5, *p* < 0.05 were considered appropriate biomarkers
Huang et al., 2021 [50]	China	Retrospective Cohort study	540 (113—intestinal necrosis group, 427—non-intestinal necrosis groups), 355:185, Mdn 5.23/7.52	NA	Most cases are idiopathic; 37 cases had pathological lead points	Fibrinogen, paletets, D-dimer	Blood tests	Pre-treatment	Determination of the risk factors for intestinal necrosis among children with failed non-surgical reduction for intussusception	Surgery	Multivariable analysis revealed that duration of symptom (OR 1.12, *p* = 0.000), fibrinogen (OR 1.26, *p* = 0.010) and D-dimer (OR 2.07, *p* = 0.000) independently predicted intestinal necrosis in individuals undergoing surgical reduction for intussusception	Intestinal necrosis in intussusception patients was more likely with D-dimer levels > 1.005 mg/L
Hou et al., 2021 [51]	China	Retrospective cohort study	564 (132—overweight and obesity group, 432—no overweight and obesity group), 405:159, <24 mth = 353, >24 mth = 211)	Ileocolic	Idiopathic (patients with identified lead points were excluded)	WBC, CRP	Blood tests	Pre-treatment	Comparison of clinical outcomes after primary air enema reduction for intussusception in grouped overweight and obese (body mass index-for-age percentile ≥ 85) pediatric patients compared with non-overweight and obese patients	Pneumatic reduction, surgical reduction	After multivariate logistic regression analysis, overweight and obesity (OR = 8.045) and WBC count ≥ 20 × 10^9^/L were risk factors for both surgical reduction (OR = 6.151) and the recurrence (OR = 3.357) of intussusception	WBC count ≥ 20 × 10^9^/L (predefined threshold)
Chen et al., 2021 [52]	China	Retrospective cohort study	115 (47—underwent intestinal resection, 68—did not undergo intestinal resection), NA, 1.79 ± 0.88 yrs/1.87 ± 0.92 yrs	NA	NA	Neutrophils, platelets, CRP, lymphocytes, albumin	Blood tests	Pre-treatment (within 2 days prior to surgery)	Investigation of the value of various combinations of inflammatory factors to predict intestinal necrosis and resection	Surgery	A combination of lymphocytic count along with C-reactive protein levels demonstrated the highest correlation with intestinal resection due to intussusception compared with other parameters in the patients, with a sensitivity of 0.82 and specificity of 0.80 for the diagnosis of strangulation	CRP > 11.26, albumin > 29.4, PLR > 188.5, LCR < 0.121, NLR > 5.72, CAR = 0.286
Zhao et al., 2021 [53]	China	Retrospective case–control study	160 (60—children with HSP, 100—control group), 71:89, mean 6.6 ± 2 yrs	Surgery: small bowel intussusception (*n* = 29), ileocolic (*n* = 19)	NA	WBC count, neutrophil count, platelet count, CRP, erythrocyte sedimentation rate, D-dimer	Blood tests	Pre-treatment	Investigation of the risk factors for intussusception in children with Henoch–Schönlein purpura	Air enema reduction, surgery	Univariate and multiple regression analyses revealed age at onset, not receiving glucocorticoid therapy within 72 h of emergence of GI symptoms, hematochezia, and increased D-dimer levels (OR = 7.193) as independent risk factors for intussusception in children with HSP (*p* < 0.05)	D-dimer levels ≥ 1 mg/L
Younes et al., 2021 [54]	South Korea	Retrospective cohort study	145 (124—pneumatic reduction, 21—surgical reduction), 95:50, mean 24.6 ± 15.2 mth	Ileocolic	Idiopathic	WBC with differential, hemoglobin, and CRP	Blood tests	At diagnosis	Identification of factors that can lead to pneumatic reduction failure in children with ileocolic intussusception	Pneumatic reduction, surgery	Multivariate analysis showed that a high segmented neutrophil count, low hemoglobin level, and higher weight percentile were significantly associated with pneumatic reduction failure	Segmented neutrophil > 67.3% hemoglobin < 12.2 g/dL
Lee et al., 2020 [55]	South Korea	Retrospective cohort study	249 (199—good outcome group, 50—poor outcome group/intussusception recurrence or required surgical reductions), 159:90, Mdn 1.8 yrs	Ileocolic	Idiopathic	pH, lactic acid, bicarbonate	Venous blood gas analysis	At diagnosis	Determination of whether lactic acid levels are associated with pediatric intussusception outcomes	Air enema, surgery	The poor and good outcome groups showed significant differences in their respective blood gas analyses for pH (7.39 vs. 7.41, *p* = 0.001), lactic acid (1.70 vs. 1.30 mmol/L, *p* < 0.001), and bicarbonate (20.70 vs. 21.80 mmol/L, *p* = 0.036). Multivariable logistic regression analyses showed that pH (OR 0.000, *p* = 0.003) and lactic acid (OR 3.066, *p* < 0.001) levels were the two factors significantly associated with poor outcomes.	When the lactic acid level cutoff values were ≥1.5, ≥2.0, ≥2.5, and ≥3.0 mmol/L, the positive predictive values for poor outcomes were 30.0, 34.6, 50.0, and 88.9%, respectively
Zhu et al., 2019 [56]	China	Retrospective cohort study	60 (acute intussusception—30/indirect inguinal hernia, control group—30), 18:12/20:10, 1.2 ± 0.6 yrs/1.3 ± 0.5 yrs	NA	NA	Vasoactive intestinal peptide, substance P (serum)	ELISA	Pre-treatment and 1, 2, and 4 weeks post-treatment	Comparison of groups, monitoring of biomarkers over time after treatment	Air enema (symptoms < 48 h, *n* = 27), surgery (abdominal distension and peritonitis, or those younger than 3 months, *n* = 3)	In a comparison of groups, the intussusception group had significantly lower serum VIP levels before treatment and significantly higher SP levels. After treatment, VIP levels gradually increased, and SP levels decreased (*p* < 0.05)	NA
Xiaolong et al., 2019 [57]	China	Retrospective cohort study	621 (62—failed group, 559—successful group), 2:1, Mdn 22 mth	Failed group: ileocolic (*n* = 58), ileoileocolic (*n* = 4)	Failed group: idiopathic (*n* = 39), polyp (*n* = 5), Meckel (*n* = 17), angioma (*n* = 1)	White blood cell counts, neutrophils, and electrolytes	Blood tests	Pre-treatment	Exploration of the risk factors associated with the failure of hydrostatic reduction	Hydrostatic reduction, surgery	Significant risk factors for failure of hydrostatic reduction in intussusception were an age of under 1-year old (OR = 3.915), duration of symptoms more than or equal to 48 h (OR = 0.056), rectal bleeding (OR = 0.283), constipation (OR = 0.086), palpable abdominal mass (OR = 0.370), and location of mass (left over right side) (OR = 13.782)	NA
Lee et al., 2019 [58]	South Korea	Retrospective cohort study	137 (23—recurrent intussusception), 80:57, mean 2.17 ± 1.36 yrs	Ileocolic (*n* = 136), small bowel type (*n* = 1)	No specific anatomical lead points were identified during surgical reduction	WBC, erythrocyte sedimentation rate, CRP, albumin	Blood tests	At diagnosis	Identification of factors related to the recurrence of intussusception in pediatric patients	Enema reduction, surgery	Patients in the recurrence group had higher levels of CRP (2.68 ± 1.49 mg/dL vs. 1.49 ± 1.03 mg/dL, *p* = 0.024). On regression analysis, age > 1 year at the time of presentation (OR = 4.79) and no history of infection (OR = 0.18) were retained as predictors of recurrence	CRP > 0.5 mg/dL
Ademuyiwa et al., 2018 [59]	Nigeria	Prospective study	75 (25—with necrotic bowel, 25—without bowel necrosis, 25—controls), 51:24, 7 ± 3.16 mth	NA	Idiopathic	Intestinal fatty-acid-binding protein (serum)	ELISA	Pre-treatment	Likelihood of bowel resection due to necrosis	Surgery (intestinal resection)	Twenty-five children were diagnosed with necrotic intussusception whose serum I-FABP immunoassay has significantly higher median compared with those without necrosis and controls (2056 ng/mL vs. 943 ng/mL and 478 ng/mL, *p* = 0.0002). Length of necrosed bowel correlates with I-FABP levels (r = 0.62)	Using a cutoff value of 1538 ng/mL, the sensitivity, specificity, PPV, and NPV were 64%, 88%, 84%, and 71%, respectively. I-FABP titer greater than 1538 ng/mL was found to have a higher likelihood of necrotic bowel (OR = 13.04, *p* = 0.002)
Lim et al., 2018 [60]	Malaysia	Retrospective observational study	172, 107:65, Mdn 2.2 yrs	NA	Failed USGHR group: lymphoma (*n* = 3), neuroendocrine tumor of the appendix (*n* = 1), Meckel’s diverticulum (*n* = 1), Henoch–Schönlein purpura hamartoma (*n* = 1), enteric duplication cyst (*n* = 1)	Hemoglobin, platelet count, and leukocytes	Blood tests	Pre-treatment	Examination of the experience and factors associated with the success or failure of ultrasound-guided hydrostatic reduction using water	USGHR, surgery	Age more than 3 years old (OR = 7.16), anemia (OR = 10.12), thrombocytosis (OR = 11.21), ultrasound findings of free fluid (OR = 9.39), and left-sided intussusception (OR = 8.18) were independently associated with USGHR irreducibility	NA
Tamas et al., 2017 [61]	USA	Prospective cohort study	39 (16—intussusception), 27:12, mean 25/20 mth	NA	NA	Lactic acid	VITROS LAC slide method	Pre-treatment	Evaluation of lactic acid levels to determine if they can predict the presence of intussusception	Enema reduction	Mean (± standard deviation) lactic acid levels were not significantly different between children with suspected (1.7 ± 0.69 mmol/L) and confirmed intussusception (1.93 ± 1.13 mmol/L) (*p* = 0.29)	NA
Carapinha et al., 2016 [62]	South Africa	Prospective observational study	97, NA, between 3 mths and 3 yrs	NA	Idiopathic	CRP	Latex immunoassay	Pre-treatment	Determination of the impact of revised protocols to better select patients for pneumatic reduction, documentation of the associated morbidity and mortality, and the factors that affect the above	Pneumatic reduction, surgery	Prolonged duration of symptoms and a raised CRP level predicted a poor outcome. A raised CRP is predictive of failure of pneumatic reduction (OR = 1.01, *p* = 0.043) and relook laparotomy (OR = 1.01, *p* = 0.025)	NA
Karabulut et al., 2010 [63]	Turkey	Case–control study	42 (22—study group, 20—control group), 28:14, mean 13 ± 5.66 mth/12.6 ± 5.1 mth	Ileocolic	Idiopathic	Hemoglobin, WBC, IL-6, CRP, BUN	Human IL-6 assay, blood tests	At diagnosis	Determination of the role of inflammation related to body mass index and atopy in the etiology of idiopathic intussusception	Enema reduction, surgery	When binary logistic regression analysis with the cutoff value of IL-6 set as 1.6 pg/mL was applied to all data, statistically significant values were obtained only when the case was in the study group and when CRP levels were increased	IL-6 = 1.6 pg/mL
Fragoso et al., 2007 [64]	Portugal	Cross-sectional study	164, 122:42, mean 11.6 ± 10.7 mth	NA	NA	WBC, neutrophils (%)	Blood tests	At diagnosis	Determination of risk factors and design and evaluation of a predictive model of air enema failure	Pneumatic reduction, manual reduction	Multivariable analysis adjusted for age and sex revealed that delayed diagnosis (evolution > 24 h) (OR = 11.52) and raised neutrophils (%) (OR = 1.06) were associated with failure	The area under the receiver operating characteristic curve was 0.826. At the best cutoff (0.15), the positive predictive value was 35% and the negative 93%. At the cutoff of 0.50, the positive predictive value was 70% and the negative 87%; the sensitivity was 29%
Willetts et al., 2001 [65]	United Kingdom	Prospective observational study	32, 23:9, Mdn 4 mth	Ileocolic	NA	Malondialdehyde, CRP, IL 6, neopterin, tumor necrosis factor alpha, endotoxin, Ig G, and IgM EndoCAb	ELISA, HPLC, immunoturbidimetric assay, colorimetric assay	pre-treatment	Investigation of selected inflammatory mediators in children with acute intussusception and to identify potentially useful plasma markers of clinical outcome	Air enema reduction, surgery	Acute levels of plasma IL-6, neopterin and CRP were significantly raised in comparison to both normal laboratory ranges and convalescent samples. Using stepwise discriminant analysis, CRP was identified as the best variable at distinguishing	NA
McDermott et al., 1994 [66]	Scotland	Retrospective cohort study	54, 32:22, 12.5 mth	Surgical findings: ileo-ileo-colic (*n* = 2), ileoileal (*n* = 1), ileocolic (*n* = 3)	Meckel diverticulum (*n* = 2), pinworm (*n* = 1)	WBC	Blood tests	Pre-treatment	Success or failure of the air enema	Air enema, surgery	The mean leucocytosis was 14,635/cubic centimeter (cc), and of the three patients with a white cell count greater than 20 000/cc, only one needed surgery. Among patients with failed reduction, leucocytosis was present in 64% (9/14)	NA
Frey & Kistler, 1994 [67]	Switzerland	Retrospective and prospective case–control study	38 (15—idiopathic intussusception group, 23 control group), NA, 9.5 ± 9.5 mth/13.5 ± 7 mth	Origin in the ileum	Idiopathic	Leucocytes, lymphocytes, reactive lymphocytes	Blood smears	At diagnosis	Investigation of whether the presence of reactive lymphocytes may guide careful supervision in atypical cases lacking classic clinical signs	Surgery	Increased absolute number of reactive lymphocytes in the infants with intussusception compared to the controls (0.14 × 10^9^/1 ± 0.08 and 0.07 × 10^9^/L ± 0.07, respectively)	NA
Reijnen et al., 1990 [68]	Netherlands	Retrospective cohort study	130—group A (*n* = 65)—hydrostatic reduction, group B (*n* = 36)—laparotomy after hydrostatic reduction had failed, group C (*n* = 21)—primary laparotomy, group D (*n* = 8)—bowel resection, 90:40, from 1 day to 14.3 yrs	Colonic components to their intussusceptions	NA	WBC	Blood tests	At diagnosis	Prediction of failure of hydrostatic reduction	Hydrostatic reduction, surgery	Rectal bleeding and duration of symptoms of more than 48 h contributed significantly to the prediction of failure of hydrostatic reduction. WBC > 20 × 10^9^/L associated with intestinal resection in univariate analysis (*p* = 0.03).	WBC > 20 × 10^9^/L (mentioned but not central to the main predictive model)—group A compared with groups B, C, and D

ELISA—enzyme-linked immunosorbent assay, VIP—vasoactive intestinal peptide, SP—substance P, tRNA—transfer ribonucleic acid, tRFs—transfer ribonucleic acid-derived fragments, qRT-PCR—quantitative reverse-transcription polymerase chain reaction, yrs—years, mth—month, OR—odds ratio, WBC—white blood cells, CRP—C-reactive protein, TBA—total bile acid, HSP—Henoch–Schonlein purpura, AI—acute intussusception, ROC—receiver operating characteristic, Mdn—median, ICU—intensive care unit, GAS—gastrin, MTL—motilin, IL—interleukin, TNF-α—tumor necrosis factor-α, ESR—erythrocyte sedimentation rate, TLC—total leukocyte counts, CBC—complete blood count. PLR—platelet-to-lymphocyte ratio, CAR—C-reactive protein/albumin ratio, NLR—neutrophil-to-lymphocyte ratio, LCR—lymphocyte-to-C-reactive protein ratio, IgAV—IgA vasculitis, CK-MB—creatine kinase-MB, I-FABP—intestinal fatty-acid-binding protein, SII—systemic immune inflammatory index, SIRI—systemic inflammation response index, PNR—platelet-to-neutrophil ratio, LMR—lymphocyte-to-monocyte ratio, alpha-GST—alpha-glutathione S-transferase, Na—sodium, K—potassium, USGHR—ultrasound-guided hydrostatic reduction, Ig—immunoglobulin, EndoCAb—antiendotoxin core antibody, HPLC—High-Performance Liquid Chromatography, MCP-1—monocyte chemoattractant protein-1, IMA—ischemia-modified albumin, BUN—blood urea nitrogen.

## Data Availability

The data that support the findings of this study are available upon request from the corresponding author.

## Supplementary Materials

The following supporting information can be downloaded at https://www.mdpi.com/article/10.3390/jcm15083114/s1, Table S1: Methodological quality of included studies according to JBI Critical Appraisal Checklist for Cohort Studies, Cross-sectional Studies, and Case–Control Studies; PRISMA 2020 Checklist.

## Abbreviations

The following abbreviations are used in this manuscript:

PRISMA	Preferred Reporting Items for Systematic Reviews and Meta-Analysis
JBI	Joanna Briggs Institute
CRP	C-reactive protein
NEC	Necrotizing enterocolitis
IBD	Inflammatory bowel disease
RNA	Ribonucleic acid
ELISA	Enzyme-linked immunosorbent assay
VIP	Vasoactive intestinal peptide
SP	Substance P
tRNA	Transfer ribonucleic acid
tRFs	Transfer ribonucleic acid-derived fragments
qRT-PCR	Quantitative reverse-transcription polymerase chain reaction
yrs	Years
mth	Month
OR	Odds ratio
WBC	White blood cells
TBA	Total bile acid
HSP	Henoch–Schönlein purpura
AI	Acute intussusception
ROC	Receiver operating characteristic
Mdn	Median
ICU	Intensive care unit
GAS	Gastrin
MTL	Motilin
IL	Interleukin
TNF-α	Tumor necrosis factor-α
ESR	Erythrocyte sedimentation rate
TLC	Total leukocyte counts
CBC	Complete blood count
PLR	Platelet-to-lymphocyte ratio
CAR	C-reactive protein/albumin ratio
NLR	Neutrophil-to-lymphocyte ratio
LCR	Lymphocyte-to-C-reactive protein ratio
IgAV	IgA vasculitis
CK-MB	Creatine kinase-MB
I-FABP	Intestinal fatty-acid-binding protein
SII	Systemic immune inflammatory index
SIRI	Systemic inflammation response index
PNR	Platelet-to-neutrophil ratio
LMR	Lymphocyte-to-monocyte ratio
alpha-GST	Alpha-glutathione S-transferase
Na	Sodium
K	Potassium
USGHR	Ultrasound-guided hydrostatic reduction
Ig	Immunoglobulin
EndoCAb	Antiendotoxin core antibody
HPLC	High-performance liquid chromatography
MCP-1	Monocyte chemoattractant protein-1
IMA	Ischemia-modified albumin
BUN	Blood urea nitrogen
