# Supplementary material for: Predictive and Prognostic Biomarkers in Pediatric Intussusception—A Systematic Review
Source: J Clin Med. 2026 Apr 19;15(8):3114. doi: 10.3390/jcm15083114 (PMC13117405; doi:10.3390/jcm15083114)
Supplement: Supplementary file 1 [file jcm-15-03114-s001.zip › jcm-4229811-Table S1.pdf]

Table S1. Methodological quality of included studies according to JBI Critical Appraisal Checklist for Cohort Studies, Cross-sectional Studies, and Case-control studies

Study	Q1	Q2	Q3	Q4	Q5	Q6	Q7	Q8	Q9	Q10	Q11	Methodological quality
Soleimanpour et al., 2025 [22]	Yes	Yes	Yes	Unclear	No	Yes	Yes	NA	NA	NA	Yes	moderate
Ammar et al., 2025 [23]	Yes	Yes	Yes	Yes	Yes	Unclear	Yes	NA	NA	NA	Yes	moderate
Kamffer et al., 2025 [24]	Yes	Yes	Yes	Yes	No	Yes	Yes	Unclear	NA	NA	Yes	moderate
Liu et al., 2025 [25]	Yes	Yes	Yes	Yes	Yes	Unclear	Yes	NA	NA	NA	Yes	moderate
Xu et al., 2025 [26]	Yes	Yes	Yes	Yes	Yes	Unclear	Yes	NA	NA	NA	Yes	moderate
Elhadidi et al., 2025 [27]	Yes	NA	NA	Unclear	No	Yes	Yes	NA	NA	NA	Yes	low
Ulusoy et al., 2025 [28]	Yes	No	Yes	Yes	Yes	Unclear	No	Yes	Yes	Yes	-	moderate
Chang et al., 2025 [29]	Unclear	Yes	Yes	Yes	Yes	Unclear	Yes	Yes	NA	NA	Yes	moderate
Tuşat & Memiş, 2025 [30]	Yes	Yes	Yes	Unclear	No	Yes	Yes	Unclear	No	NA	Yes	moderate

Yu et al., 2024 [31]	Yes	Yes	Yes	Unclear	Yes	Yes	Yes	Yes	Yes	NA	Yes	high
Xia et al., 2024 [32]	Yes	Yes	Yes	Yes	Yes	Yes	Yes	NA	NA	NA	Yes	moderate
Wei et al., 2024 [33]	Yes	Yes	Yes	Unclear	No	Yes	Yes	Yes	NA	NA	Yes	moderate
Shah et al., 2024 [34]	Yes	Yes	Yes	No	No	Unclear	Yes	Yes	NA	NA	Yes	moderate
Budiananti et al., 2024 [35]	Yes	Yes	Yes	Yes	No	No	Yes	Yes	-	-	-	high
Mu et al., 2024 [36]	NA	NA	NA	Unclear	No	NA	Yes	Unclear	No	No	Yes	low
Liu et al., 2024 [37]	Yes	Yes	Yes	Unclear	Yes	Unclear	Yes	NA	NA	NA	Yes	moderate
Liu et al., 2024 [38]	Yes	NA	NA	Yes	Yes	Yes	Yes	Yes	No	NA	Yes	moderate
Mu, 2024 [39]	Yes	Unclear	Yes	Yes	Yes	Yes	Yes	Yes	Yes	Yes	Yes	high
Kocaoğlu et al., 2024 [40]	Yes	Yes	Unclear	Yes	Unclear	Unclear	No	Yes	Yes	Yes	-	moderate
Yu et al., 2023 [41]	Yes	Yes	Yes	Unclear	Yes	Yes	Yes	Unclear	NA	NA	Yes	moderate

Yang et al., 2023 [42]	Yes	Yes	Yes	Yes	Yes	Yes	Yes	Yes	NA	NA	Yes	high
Zhuang et al., 2023 [43]	Yes	Yes	Yes	Yes	Yes	Unclear	Yes	NA	NA	NA	Yes	moderate
Delgado-Miguel et al., 2023 [44]	Yes	Unclear	Yes	Yes	Yes	Unclear	No	Yes	Yes	Yes	-	moderate
Zhang et al., 2023 [45]	Yes	Yes	Yes	Yes	Yes	Yes	Yes	Yes	NA	NA	Yes	high
Wu et al., 2022 [46]	Yes	Yes	Yes	Yes	Yes	NA	Yes	Yes	NA	NA	Yes	moderate
Liu et al., 2022 [47]	Yes	Yes	Yes	Yes	Yes	Unclear	Yes	NA	NA	NA	Yes	moderate
Zhu et al., 2022 [48]	Yes	Yes	Yes	Yes	Yes	Yes	Yes	Unclear	NA	NA	Yes	moderate
Zhao et al., 2021 [49]	Yes	Yes	Yes	Yes	Unclear	No	Yes	Yes	-	-	-	high
Huang et al., 2021 [50]	Yes	Yes	Yes	Yes	Yes	Unclear	Yes	NA	NA	NA	Yes	moderate
Hou et al., 2021 [51]	Yes	Yes	Yes	Yes	Yes	Unclear	Yes	Yes	No	NA	Yes	moderate

Chen et al., 2021 [52]	Yes	Yes	Yes	Unclear	No	Yes	Yes	NA	NA	NA	Yes	moderate
Zhao et al., 2021 [53]	Yes	No	Yes	Yes	Yes	No	Yes	Yes	Yes	Yes	-	high
Younes et al., 2021 [54]	Yes	Yes	Yes	Unclear	No	Yes	Yes	Yes	Yes	NA	Yes	moderate
Lee et al., 2020 [55]	Yes	Yes	Yes	Unclear	NA	Yes	Yes	Yes	NA	NA	Yes	moderate
Zhu et al., 2019 [56]	Yes	Yes	Yes	NA	No	Yes	Yes	Yes	Yes	No	Yes	moderate
Xiaolong et al., 2019 [57]	Yes	Yes	Yes	Unclear	No	Yes	Yes	Yes	Yes	No	Yes	moderate
Lee et al., 2019 [58]	Yes	Yes	Yes	Unclear	No	Yes	Yes	NA	NA	NA	Yes	moderate
Ademuyiwa et al., 2018 [59]	Yes	Yes	Yes	No	No	Unclear	Yes	NA	NA	NA	Yes	low
Lim et al., 2018 [60]	Yes	Yes	Yes	Yes	Yes	NA	Yes	Unclear	NA	NA	Yes	moderate

Tamas et al., 2017 [61]	Yes	Yes	Yes	Unclear	No	Unclear	Yes	Yes	Yes	NA	Yes	moderate
Carapinha et al., 2016 [62]	Yes	Unclear	Yes	No	No	Yes	Yes	Unclear	No	No	Yes	low
Karabulut et al., 2010 [63]	Yes	Yes	Yes	Yes	Yes	Unclear	No	Yes	NA	Yes	-	moderate
Fragoso et al., 2007 [64]	Yes	Yes	Yes	Yes	Unclear	Yes	Yes	Yes	-	-	-	high
Willetts et al., 2001 [65]	Yes	Unclear	Yes	Unclear	No	Yes	Yes	Yes	Yes	No	Yes	moderate
McDermott et al., 1994 [66]	Yes	NA	NA	Unclear	No	Yes	Yes	Yes	Yes	NA	Yes	moderate
Frey & Kistler, 1994 [67]	Unclear	No	Unclear	Yes	Yes	No	No	Yes	NA	Yes	-	low
Reijnen et al., 1990 [68]	Yes	Yes	Yes	Unclear	No	NA	Yes	No	NA	NA	Yes	low

Questions for cohort studies: Q1 = Were the two groups similar and recruited from the same population?, Q2 = Were the exposures measured similarly to assign people to both exposed and unexposed groups?, Q3 = Was the exposure measured in a valid and reliable way?, Q4 = Were confounding factors identified?, Q5 = Were strategies to deal with confounding factors stated?, Q6 = Were the groups/participants free of the outcome at the start of the study (or at the moment of exposure)?, Q7 = Were the outcomes measured in a valid and reliable way?, Q8 = Was the follow up time reported and sufficient to be long enough for outcomes to occur?, Q9 = Was follow up complete, and if not, were the reasons to loss to follow up described and explored?, Q10 = Were strategies to address incomplete follow up utilized?, Q11 = Was appropriate statistical analysis used?

Questions for analytical cross-sectional studies: Q1 = Were the criteria for inclusion in the sample clearly defined?, Q2 = Were the study subjects and the setting described in detail?, Q3 = Was the exposure measured in a valid and reliable way?, Q4 = Were objective, standard criteria used for measurement of the condition?, Q5 = Were confounding factors identified?, Q6 = Were strategies to deal with confounding factors stated?, Q7 = Were the outcomes measured in a valid and reliable way?, Q8 = Was appropriate statistical analysis used?
Questions for case control studies: Q1 = Were the groups comparable other than the presence of disease in cases or the absence of disease in controls?, Q2 = Were cases and controls matched appropriately?, Q3 = Were the same criteria used for identification of cases and controls?, Q4 = Was exposure measured in a standard, valid and reliable way?, Q5 = Was exposure measured in the same way for cases and controls?, Q6 = Were confounding factors identified?, Q7 = Were strategies to deal with confounding factors stated?, Q8 = Were outcomes assessed in a standard, valid and reliable way for cases and controls?, Q9 = Was the exposure period of interest long enough to be meaningful?, Q10 = Was appropriate statistical analysis used?
Answers: Yes, No, Unclear or NA (Not/Applicable)
Based on this percentage, the methodological quality of each study was classified as low (<50%), moderate (50–74%), or high (>75%).

References

22. Soleimanpour Z, Memarian S, Rajabi MM, Zamani Z, Alimadadi H, Gharib B. Clinical and paraclinical differences between pediatric patients requiring surgical versus non-surgical treatment for intussusception: A retrospective study at a referral center in Iran. BMC Surg. 2025, 25, 373.
23. Ammar S, Sellami I, Krichen E, Rhaiem W, Sellami S, Hbaieb M, Kammoun M, Jarraya A, Zitouni H, Mhiri R. Predicting surgical intervention in pediatric intussusception using machine learning model. Tunis Med. 2025, 103, 792-797.
24. Kamffer CM, du Preez H, van Rensburg JJ. Risk factors associated with the outcomes of fluoroscopy-guided pneumatic enema reductions of intussusceptions. SA J. Radiol. 2025, 29, 3155.
25. Liu D, Cai H, He QQ, Wang S, Li F, Zhang J, Fu YJ, Su ZL, Jiang YD, Jing L. Systemic immune inflammatory index (SII) as a predictive marker for the failure of air enema treatment in children with Ileocolic intussusception: a case-control study. BMC Pediatr. 2025, 26, 92.
26. Xu XX, Cai YJ, Liu J, Zhuang Y, Ma Q. Predicting bowel necrosis in pediatric acute intussusception using roundness and other related factors. BMC Pediatr. 2025, 25, 843.
27. Elhadidi M, Elghazaly M, El-Saied AW, Awad M, Elayyouti M. Bypassing the Delay: Directing Pediatric Intussusception Cases to the OR Through Inflammatory Marker Assessment. World J. Surg. 2025, 49, 1519-1525.
28. Ulusoy E, Ulusoy O, Sütçüoğlu N, Küme T, Şık N, Yılmaz D, Duman M. Is it possible to predict the spontaneous reduction of pediatric intussusception using biomarkers? Pediatr. Int. 2025, 67, e70141.
29. Chang CY, Chen SC, Kuo HC, Huo CH, Tsai CF, Yang HY. Beyond hyponatremia: The crucial role of hypernatremia and hypokalemia in pediatric intussusception surgical outcomes. J. Pediatr. Surg. 2025, 162835.
30. Tuşat M, Memiş S. Evaluation of inflammatory markers and HALP score in childhood intussusceptions. Annals of Clinical and Analytical Medicine 2025, 16, 530-534.

31. Yu YY, Zhang JJ, Xu YT, Lin ZX, Guo SK, Li ZR, Huang HY, Huang XZ. Developing and validating a nomogram for early predicting the need for intestinal resection in pediatric intussusception. Front. Pediatr. 2024, 12, 1409046.
32. Xia B, Chen G, Liu Q, Yan C, Lu P, Guo C. A comprehensive scoring system for the better prediction of bowel resection in pediatric intussusception. BMC Gastroenterol. 2024, 24, 180.
33. Wei XY, Huo HC, Li X, Sun SL, Zhang J. Relationship between postoperative rehabilitation style, gastrointestinal function, and inflammatory factor levels in children with intussusception. World J. Gastrointest. Surg. 2024, 16, 2640-2648.
34. Shah JY, Banday I, Hamdani HZ, Haq MF, Parray FQ, Banday M, Bhat GA. A Study of Predictors of Failure of Nonoperative Management of Ileocolic Intussusception in Children. Euroasian J. Hepatogastroenterol. 2024, 14, 81-85.
35. Budiananti A, Hariastawa IA, Matulatan F. Correlation between pathology indicators and intestinal necrosis in pediatric intussusception. Chirurgia (Turin) 2024, 37, 183-187.
36. Mu J, Hao J, Zhen X, Mo X, Sheng Y. Surgical complications in children with IgA vasculitis: clinical analysis of 28 cases. Investigación Clínica 2024, 65, 134-142.
37. Liu J, Wang Y, Jiang Z, Duan G, Mao X, Zeng D. Developing a Nomogram for Predicting Surgical Intervention in Pediatric Intussusception After Pneumatic Reduction: A Multicenter Study from China. Ther. Clin. Risk. Manag. 2024, 20, 313-323.
38. Liu J, Zeng D, Jiang Z, Xiu W, Mao X, Li H. Developing a nomogram to predict recurrent intussusception after pneumatic reduction of primary intussusception in children. BMC Surg. 2024, 24, 275.
39. Mu J. Clinical characteristics and risk factors of IgA vasculitis with intussusception and intestinal perforation. J. Paediatr. Child Health. 2024, 60, 5-11.
40. Kocaoğlu Ç, Kocaoğlu C, Madenci H. Can Ischemia-Modified Albumin Be a Helpful Marker in the Diagnosis and Follow-Up of Childhood Intussusception? Pediatr. Emerg. Care. 2024, 40, e105-e107.
41. Yu S, Feng W, Wang Y, Zhao M, Tu Y, Guo Z. Serum total bile acid levels assist in the prediction of acute intussusception with abdominal type Henoch-Schonlein purpura in children. Front. Pediatr. 2023, 11, 1183470.
42. Yang M, Xie Y, Zhuang Y, Chen Y, Lin X, Liu Z, Zhang P, Xiao W, Chen Y, Chen C, Zheng L, Duan S. Risk factors and predictive models for early recurrent intussusception in children: a retrospective cohort study. Transl. Pediatr. 2023, 12, 1800-1809.
43. Zhuang Y, Wang X, Fan X, Li F, He G, Luo M, Tang Y. Developing a nomogram for predicting surgical intervention in pediatric intussusception after hydrostatic reduction. Front. Pediatr. 2023, 11, 1092548.
44. Delgado-Miguel C, García A, Delgado B, Muñoz-Serrano AJ, Miguel-Ferrero M, Camps J, Lopez-Santamaria M, Martinez L. Neutrophil-to-Lymphocyte Ratio as a Predictor of the Need for Surgical Treatment in Children's Intussusception. Eur. J. Pediatr. Surg. 2023, 33, 422-427.
45. Zhang J, Dong Q, Su X, Long J. Factors associated with in-hospital recurrence of intestinal intussusception in children. BMC Pediatr. 2023, 23, 428.
46. Wu TH, Huang GS, Wu CT, Lai JY, Chen CC, Hu MH. Clinical characteristics of pediatric intussusception and predictors of bowel resection in affected patients. Front. Surg. 2022, 9, 926089.
47. Liu Z, Yu M, Li N, Hu M, Su F, Hong L, Deng X, Zhang S, Tao Q. Predictors of Surgical Treatment of Intussusception and Intussusception Complicated with Intestinal Necrosis in Children. Inn. J. Pediatr. 2022, 32, e117708.

48. Zhu D, Xu X, Zhang M, Wang T, Zhu H. Significance of MCP-1 in predicting the short-term recurrence of primary intussusception in children: An observational study. Medicine (Baltimore). 2022, 101, e30743.
49. Zhao L, Chen W, Guo WL. Serum tRNA-derived fragments as potential biomarkers in children with acute intussusception. Arch. Med. Sci. 2021, 19, 1889-1900.
50. Huang HY, Lin XK, Guo SK, Bao XZ, Lin ZX, Li ZR, Huang XZ. Haemostatic indexes for predicting intestinal necrosis in children with intussusception. ANZ J. Surg. 2021, 91, 1485-1490.
51. Hou J, Hou J, Die X, Sun J, Zhang M, Liu W, Wang Y. Impact of overweight/obesity on clinical outcomes after reduction for intussusception in children. Pediatr. Surg. Int. 2021, 37, 887-895.
52. Chen B, Cao J, Yan C, Zheng C, Chen J, Guo C. A promising new predictive factor for detecting bowel resection in childhood intussusception: the lymphocyte-C-reactive protein ratio. BMC Pediatr. 2021, 21, 577.
53. Zhao Q, Yang Y, He SW, Wang XT, Liu C. Risk factors for intussusception in children with Henoch-Schönlein purpura: A case-control study. World J. Clin. Cases. 2021, 9, 6244-6253.
54. Younes A, Lee S, Lee JI, Seo JM, Jung SM. Factors Associated with Failure of Pneumatic Reduction in Children with Ileocolic Intussusception. Children (Basel). 2021, 8, 136.
55. Lee JY, Byun YH, Park JS, Lee JS, Ryu JM, Choi SJ. Lactic acid level as an outcome predictor in pediatric patients with intussusception in the emergency department. BMC Pediatr. 2020, 20, 184.
56. Zhu ZC, Wang HB, Yan XQ, Yang J, Duan XF, Bian HQ, Kuang HF. Serum substance P and vasoactive intestinal peptide levels in infants with acute intussusception. J. Int. Med. Res. 2019, 47, 2446-2451.
57. Xiaolong X, Yang W, Qi W, Yiyang Z, Bo X. Risk factors for failure of hydrostatic reduction of intussusception in pediatric patients: A retrospective study. Medicine (Baltimore). 2019, 98, e13826.
58. Lee DH, Kim SJ, Lee HJ, Jang HJ. Identifying Predictive Factors for the Recurrence of Pediatric Intussusception. Pediatr. Gastroenterol. Hepatol. Nutr. 2019, 22, 142-151.
59. Ademuyiwa A, Alakaloko F, Elebute O, Bode C, Udenze I. Serum intestinal fatty-acid binding protein: predictor of bowel necrosis in pediatric intussusception. J. Pediatr. Surg. 2018, 53, 335-338.
60. Lim RZM, Lee T, Ng JYZ, Quek KF, Abdul Wahab N, Amansah SL, Vellusamy VMAM, Ngim CF. Factors associated with ultrasound-guided water enema reduction for pediatric intussusception in resource-limited setting: potential predictive role of thrombocytosis and anemia. J Pediatr Surg. 2018, 53, 2312-2317.
61. Tamas V, Ishimine P. Comparison of Lactic Acid Levels in Children with Suspected and Confirmed Intussusception. J. Emerg. Med. 2017, 53, 815-818.
62. Carapinha C, Truter M, Bentley A, Welthagen A, Loveland J. Factors determining clinical outcomes in intussusception in the developing world: Experience from Johannesburg, South Africa. S. Afr. Med. J. 2016, 106, 177-180.
63. Karabulut B, Erdoğan D, Bostancı I, Onde U, Karakoç AE. Are interleukin-6, body mass index and atopy crucial in infantile intussusception? Indian J. Pediatr. 2010, 77, 1257-1260.
64. Fragoso AC, Campos M, Tavares C, Costa-Pereira A, Estevão-Costa J. Pneumatic reduction of childhood intussusception. Is prediction of failure important? J. Pediatr. Surg. 2007, 42, 1504-1508.

65. Willetts IE, Kite P, Barclay GR, Banks RE, Rumley A, Allgar V, Stringer MD. Endotoxin, cytokines and lipid peroxides in children with intussusception. Br. J. Surg. 2001, 88, 878-883.
66. McDermott VG, Taylor T, Mackenzie S, Hendry GM. Pneumatic reduction of intussusception: clinical experience and factors affecting outcome. Clin. Radiol. 1994, 49, 30-34.
67. Frey B, Kistler W. Reactive lymphocytes and intussusception in childhood. Pediatr. Surg. Int. 1994, 9, 360-361.
68. Reijnen JA, Festen C, van Roosmalen RP. Intussusception: factors related to treatment. Arch. Dis. Child. 1990, 65, 871-873.
